# Weld Seam Tracking and Detection Robot Based on Artificial Intelligence Technology

**DOI:** 10.3390/s23156725

**Published:** 2023-07-27

**Authors:** Jiuxin Wang, Lei Huang, Jiahui Yao, Man Liu, Yurong Du, Minghu Zhao, Yaoheng Su, Dingze Lu

**Affiliations:** 1 School of Science, Xi′an Polytechnic University, Xi′an 710048, Chinaludingze@whu.edu.cn (D.L.); 2 School of Mechatronic Engineering and Automation, Shanghai University, Shanghai 200444, China

**Keywords:** inspection robot, DeepLabv3+, weld seam identification, wall-climbing robot

## Abstract

The regular detection of weld seams in large-scale special equipment is crucial for improving safety and efficiency, and this can be achieved effectively through the use of weld seam tracking and detection robots. In this study, a wall-climbing robot with integrated seam tracking and detection was designed, and the wall climbing function was realized via a permanent magnet array and a Mecanum wheel. The function of weld seam tracking and detection was realized using a DeepLabv3+ semantic segmentation model. Several optimizations were implemented to enhance the deployment of the DeepLabv3+ semantic segmentation model on embedded devices. Mobilenetv2 was used to replace the feature extraction network of the original model, and the convolutional block attention module attention mechanism was introduced into the encoder module. All traditional 3×3 convolutions were substituted with depthwise separable dilated convolutions. Subsequently, the welding path was fitted using the least squares method based on the segmentation results. The experimental results showed that the volume of the improved model was reduced by 92.9%, only being 21.8 Mb. The average precision reached 98.5%, surpassing the original model by 1.4%. The reasoning speed was accelerated to 21 frames/s, satisfying the real-time requirements of industrial detection. The detection robot successfully realizes the autonomous identification and tracking of weld seams. This study remarkably contributes to the development of automatic and intelligent weld seam detection technologies.

## 1. Introduction

With the rapid development of industrial technology, large-scale welding devices, such as pressure vessels and conduits, have been widely used in the energy industry. However, these devices are susceptible to weld seam damages or cracks, which, if left undetected, can escalate into serious safety hazards [1]. Manual inspection, the current predominant method for weld seam detection, faces limitations in terms of reliability and safety in line with modern manufacturing standards. Therefore, the development of weld seam tracking robots has drawn considerable attention in the field of nondestructive detection [2].

Welding seam tracking and detection robots can complete the task of welding seam detection of bridges, pipelines and storage tanks in minutes [3,4,5], and welding seam tracking and detection robots can not only effectively avoid the working environment that may bring health problems to workers but also consistently ensure a high quality of weld tracking detection [6]. A weld-tracking robot based on the real-time measurement of different grades of welds is proposed. A combined structure with a laser rangefinder, light detection and ranging and depth camera is installed on the rolling platform, which can effectively improve the coverage of weld tracking detection [7]. Based on the six-axis robot arm seam tracking experimental platform, a seam tracking detection robot is designed, which can realize stable seam tracking detection under external interference [8]. A spherical tank inspection robot system is proposed, which can identify and track the weld on the shortest running route based on the visual sensor and artificial intelligence algorithm [9].

Large-scale welding devices often require high-altitude detection, posing technical challenges for weld seam tracking and detection robots, particularly in terms of wall-climbing functionality. Extensive research has been conducted in this area, focusing on magnetic wall-climbing robots [10,11,12] and negative-pressure wall-climbing robots [13,14,15]. Negative-pressure wall-climbing robots rely on flatness for effective adsorption, making them more suitable for smooth walls. However, large weldments exhibit diverse structures and weld seams, posing remarkable challenges to the adsorption capacity of negative-pressure wall-climbing robots. In addition, permanent magnet adsorption or electromagnetic adsorption methods offer more pronounced advantages over negative-pressure adsorption because large weldments primarily consist of ferromagnetic materials.

Weld seam tracking and detection robots must achieve a stable mobile platform in complex environments and possess accurate and efficient capabilities for weld seam feature and path extraction. Optical sensors and image processing algorithms are commonly utilized for this purpose [16]. A visual detection system based on structured light and an image processing algorithm employing laser contour feature points have been used to measure the sizes of different weld seams, enabling defect detection based on visual characteristics [17]. The Harris corner detector and the Canny operator have been applied to eliminate the influence of natural light on weld seam feature extraction, making them suitable for differently shaped weld seams [18]. The Gabor filtering algorithm has been adopted to extract weld seam features from images, followed by threshold segmentation to obtain weld seam contours and edges [19]. An image preprocessing method based on the Sobel filtering algorithm and a noise reduction method based on dilation and erosion operations are proposed to realize the autonomous detection of differently shaped welding paths, achieving an error rate within 2.6% [20]. The weld seam identification process is divided into two stages to enable the accurate identification of weld trajectories under varying lighting conditions. The first stage employs an image preprocessing algorithm based on illumination correction to eliminate the influence of illumination. In the second stage, an image extraction algorithm based on iterative threshold segmentation and morphological processing is proposed to obtain continuous binary images of weld seams. The central point extraction algorithm and polynomials based on the least squares method are then applied to obtain the fitted curve of the weld seam center [21].

Although the above methods partially capture the characteristics of weld seams, the complex environment surrounding weld seams, including surface rust and corrosion, can adversely affect the accuracy and speed of traditional algorithms. With the development of deep learning, neural networks have found widespread application in object detection [22,23,24]. A novel approach for automatic weld seam detection and identification based on the deep convolutional neural network (DCNN) model is proposed, involving the training of two network models. The generative adversarial network is used to generate high-quality training samples, and the DCNN network is utilized for weld seam identification and localization, achieving an average precision of 91.02% [25]. Given the insufficient weld seam training samples, a data augmentation method based on a deep semantic segmentation network is employed to expand the dataset, which is then used to train the lightweight YOLOv3 model, enabling the rapid localization of weld trajectories [26]. A weld seam detection and target recognition algorithm based on YOLOv5 is proposed for complex environments to ensure cost-effective computation, achieving a recognition accuracy exceeding 90% and improving operational efficiency by 12% [27]. A weld seam instance segmentation algorithm based on Mask R-CNN is proposed to address the influence of target detection on weld seam tracking accuracy, demonstrating a single-path planning time of 180 ms and an average precision of 67.6% [28].

Despite the progress made in the aforementioned studies toward achieving weld seam tracking and detection, certain issues persist. The algorithm’s recognition accuracy remains seriously affected by the complex environment of weld seam detection robots. Although the above research primarily focuses on seam tracking and detection functionality, several challenges remain, particularly regarding the complex environment’s influence on algorithmic recognition accuracy. Given that algorithms are executed on the robot’s hardware equipment, path planning algorithms should prioritize minimizing computational cost. Consequently, this study aims to address these concerns by (1) establishing comprehensive weld seam datasets through data augmentation and (2) enhancing the DeepLabv3+ semantic segmentation model to realize weld seam tracking and detection with low computational costs, minimal time delays, and high accuracy.

## 2. Implementation of the Weld Seam Tracking and Detection Robot System

### 2.1. Mechanical Design of the Robot

**Figure 1:**
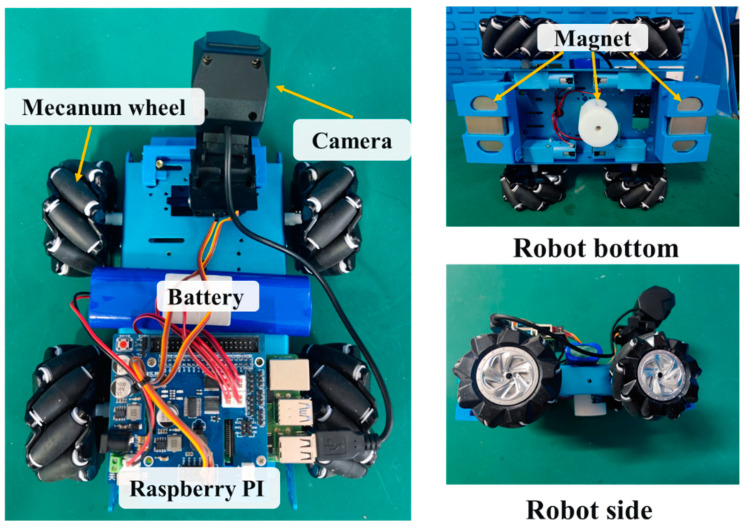
Weld seam tracking and detection robot.

In this study, a weld seam detection and tracking robot was developed, as shown in Figure 1, to realize stable adsorption and movement performance for weld seam tracking and detection on the wall. The design of the wall-climbing robot’s adsorption and movement modes needed to meet higher requirements to accommodate wall corrosion and unevenness.

According to the measurement and functional requirements, a wall-climbing detection robot for seam identification and tracking is designed. The detection robot consists of four McNamham wheels, four DC motors, an adsorption module, a raspberry pie and a robot body frame. A camera is installed at the front of the robot to collect weld images in real time, an adsorption module is installed at the bottom for the robot to climb the wall, and a raspberry pie is installed at the top for the deployment and implementation of the neural network algorithm at the embedded end. Four motors are installed around the robot frame to control the four wheels of the car body, respectively, so as to control the movement speed and direction.

A wheeled movement mode was chosen to ensure the safety of the wall-climbing robot during movement on the wall and prevent slipping or falling hazards. This mode offered flexibility and strong obstacle crossing capabilities. The robot can move in different directions and steer at various angles by adjusting the wheel speeds on both sides, allowing it to adapt to different weld seam surfaces.

For safe crawling on vertical or angled wall surfaces, the wall-climbing robot required a stable adsorption force, necessitating the design and selection of an appropriate adsorption device. Existing wall-climbing robots employ three main adsorption methods: negative-pressure adsorption, magnet adsorption, and bionic adsorption. In this study, the permanent magnet adsorption method was adopted due to its simpler structural design and more reliable adsorption force compared to those of the other two methods.

The adsorption magnet used in this study was an industrial-grade permanent magnet, specifically a rubidium magnet made of rubidium iron boron, NdFeB. The performance parameters of the magnet are shown in Table 1. Both circular and rectangular permanent magnets were selected to ensure stable adsorption capacity.

**Figure 2:**
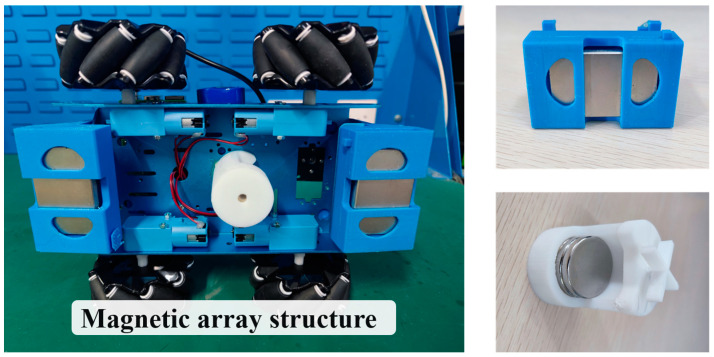
Permanent magnet array structure.

In the process of weld seam detection and tracking, the detection robot operates in two states: the normal climbing state and obstacle-crossing state. Obstacle crossing ability is an important performance index of the robot because it needs to cross weld seams during operation. The height of the weld seam is approximately 3–4 mm. A magnetic array structure was designed in this study to enhance the robot’s ability to cross obstacles and maintain a self-adaptive adsorption force while turning. The structure was equipped with different numbers of adsorption magnets in front of the car body, behind the car body, and on the belly of the car body. Figure 2 depicts the permanent magnet array structure, which includes two permanent magnet structures at the front and rear. Inside, two circular magnets and a cuboid magnet were overlaid. The magnet modules in the belly of the car body were composed of four circular magnets. The front and rear ends of the car body were equipped with superposed magnets to increase the adsorption capacity by realizing the superposition of magnetic fields. The magnets in the belly of the car body were used to maintain stability during normal detection and obstacle-crossing.

In practical applications, achieving continuous changes in the magnetizing direction of permanent magnets is not feasible. This study employed discrete permanent magnets spliced together in an approximately sinusoidal magnetizing situation. Under this configuration, the magnetic induction intensity (z > 0) on the strong magnetic field side of the linear permanent magnet array can be expressed by Formula (1),

(1) $$\{\begin{array}{c} B_{0}=B_{r}\left(1-e^{-kd}\right)\sin(\pi /m)/(\pi /m)\\ B_{y}=B_{0}e^{-kz}\sin ky\\ B_{z}=B_{0}e^{-kz}\cos ky \end{array}$$

where *m* represents the number of magnetic blocks in each wavelength; $B_{0}$ denotes the surface magnetic induction intensity of the strong magnetic field side of the magnet, where the greater the value of *m* is, the closer $B_{0}$ is to the ideal value; $B_{r}$ is the magnetic induction intensity. $B_{y}$ and $B_{z}$ represent the components of the magnetic induction intensity in the y and z directions, respectively; k is the component of wave vector in z direction; d is the distance between the permanent magnet and the ground; y and *z* are the components of the relative position in the direction of y and z coordinate axes, respectively.

The magnetic field force of the magnet was obtained on the basis of the gap between the independent magnet and the wall surface. Assuming the magnet was magnetized in one direction along the long edge, the magnetic force can be approximated using Formula (2),

(2) $$F=\frac{\mu_{r}-1}{2\mu_{0}\mu_{r}}B^{2}S$$

where $\mu_{r}$ is the relative permeability; $\mu_{0}$ is permeability of vacuum; B is the magnetic induction intensity; S is the area of the plane perpendicular to the magnetic field direction.

The performance parameters of the weld seam-tracking robot are displayed in Table 2. The robot had a dead weight ($M_{G}$) of 1.70 kg and a maximum payload capacity ($M_{l}$) of 1.96 kg. The robot obtained an adsorption force (F) of 366.12 N and an obstacle crossing height ($h_{m}$) of 11 mm due to the improved wheeled movement mode and the design of the adsorption structure. Its maximum climbing speed ($V_{max}$) was set to 0.2 m/s, and it had a maximum continuous working time (T) of approximately 100 min. The robot had a sufficient adsorption force, obstacle crossing height, and endurance capacity for wall movement as determined through the analysis of the performance parameters. Improving the performance of the weld seam-tracking robot can expand its application range and reduce operational risks.

### 2.2. System Composition

**Figure 3:**
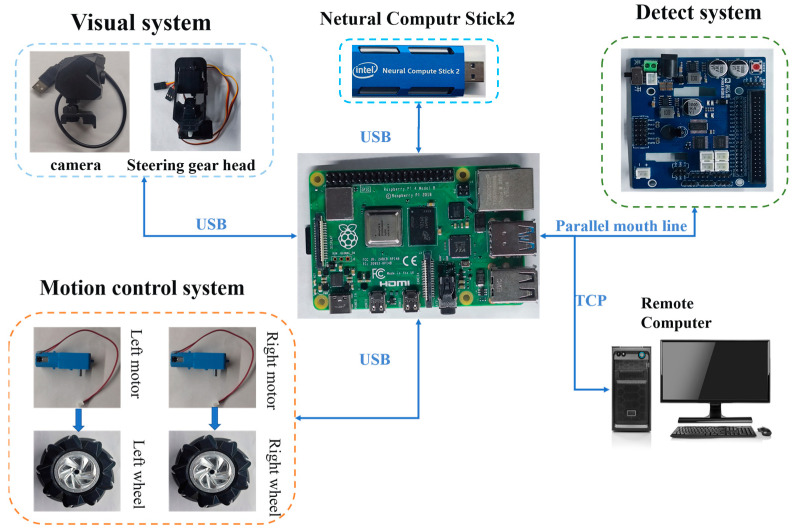
Robot hardware diagram.

The weld seam recognition and tracking system of the seam-tracking and detection robot consisted of multiple components, including seam recognition, path fitting, motion control, and data transmission. As shown in Figure 3, the robot system included a visual system, a seam tracking and detection system, a motion control system, and a remote computer. The visual system comprised an industrial camera and a two-degrees-of-freedom steering platform, enabling the comprehensive acquisition of weld seam images. The motion control system consisted of the main controller (Raspberry Pi), a DC motor, a Mecanum wheel, and a remote-control unit, which facilitated motor functions and remote control in conjunction with the remote computer. The weld seam detection system was used to locate the position information of the weld seam, and the image preprocessing subsystem reduced the computational load of the system while enhancing image quality. The path fitting system was used to fit the centerline of discrete information points, enabling the robot to navigate along the weld path. The robot designed in this study addressed the following challenges:

(1) Achieving stable operation on the steel plate wall surfaces using the Mecanum wheel in combination with the permanent magnet array;
(2) Identifying and extracting weld seams through the weld seam recognition system;
(3) Tracking weld seams by fitting the welding path.

## 3. Design of the Weld Recognition and Tracking Algorithm

### 3.1. Establishment of Weld Seam Datasets

#### 3.1.1. Construction of Initial Weld Seam Datasets

**Figure 4:**
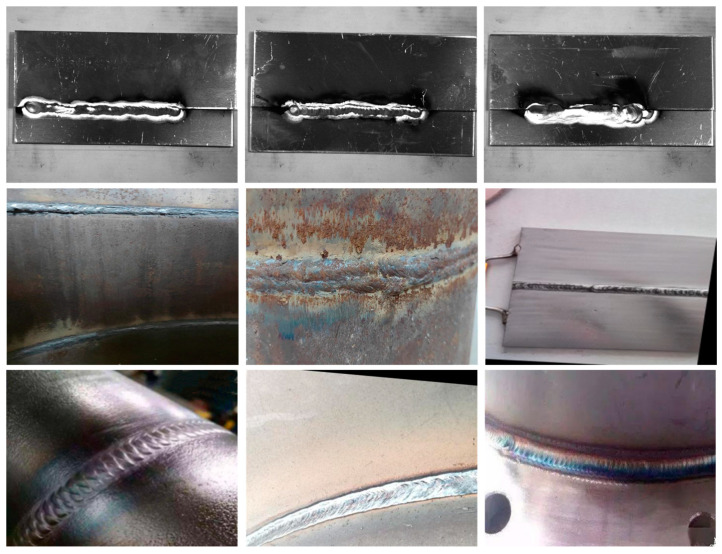
Examples of dataset samples.

The weld seam detection algorithm used in this study was the DeepLabv3+ semantic segmentation network model based on deep learning. The weld seams needed to be segmented from the background image through region segmentation to annotate the weld seam image datasets and meet the requirements of the semantic segmentation network model. A self-built dataset consisting of 776 weld seam images was used, which were obtained from three different datasets, as shown in Figure 4. First, 300 open-source datasets with consistency, a background, and environment were downloaded from the Kaggle data analysis platform [29]. Additionally, 300 real weld seam images, varying in environment, background, and size, were collected in the laboratory environment. Finally, 176 images were obtained via crawling online using Python-based crawler technology.

#### 3.1.2. Augmentation of Weld Seam Data

Both offline and online data augmentation strategies are used in this study. Offline data augmentation strategies are carried out before network training, and online data augmentation strategies are carried out during data set reading after network training.

**Figure 5:**
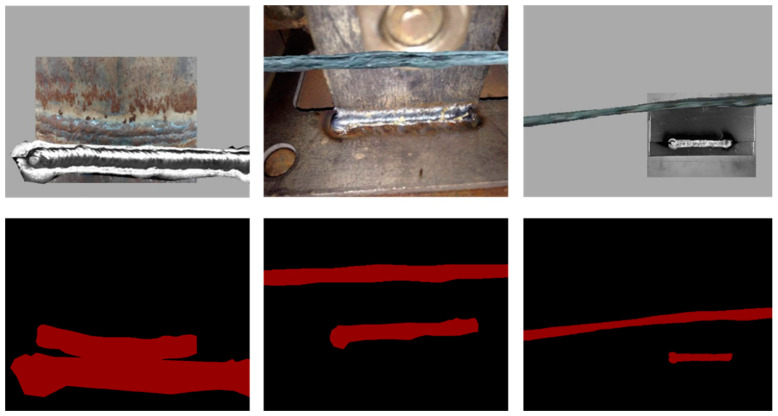
Copy and paste data augmentation results.

In deep learning, the task of semantic segmentation is to extract and segment the semantic information of the target image. When training samples, network models are prone to overfitting when the data set size is extremely small. Therefore, an offline data augmentation strategy was performed in this study using the copy paste [30] method. The original 776 weld seam images and their corresponding mask images were simultaneously subjected to data augmentation. The data augmentation results are shown in Figure 5. The 776 augmented new weld seam images were mixed with the original weld seam images, resulting in a new dataset containing 1552 weld images. The corresponding label dataset was also expanded to 1552 images, effectively preventing model overfitting during training and increasing the data size for model training. In addition, the online data expansion strategy adopts a new data enhancement strategy to randomly generate new data graphs during training, including brightness adjustment, image cropping, image flipping and filtering for denoising.

**Figure 6:**
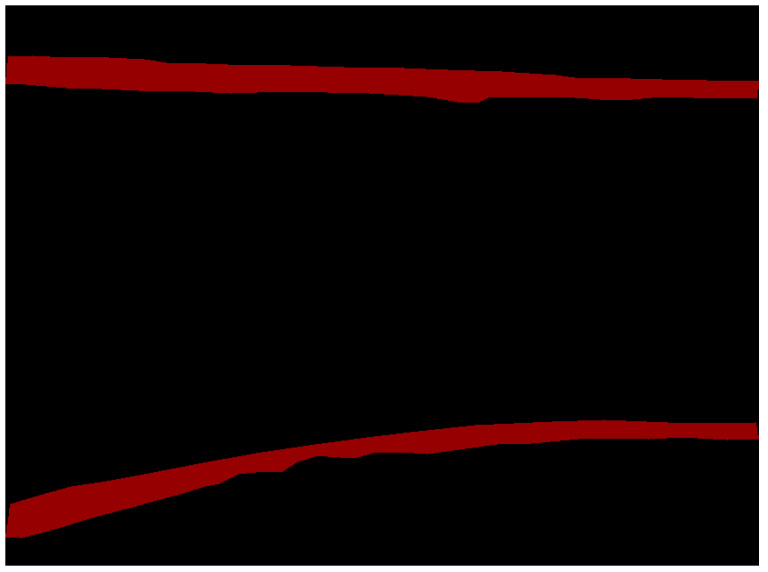
Label example.

The dataset was divided into a 7:3 proportion to obtain the original image dataset. Subsequently, the dataset was annotated by segmenting the weld seam from the background image using region segmentation. In this study, the Labelme image annotation tool was employed to annotate the weld seam images and obtain labeled sample data for the dataset, as shown in Figure 6.

### 3.2. Establishment of a Semantic Segmentation Model

#### 3.2.1. DeepLabv3+Basic Model

**Figure 7:**
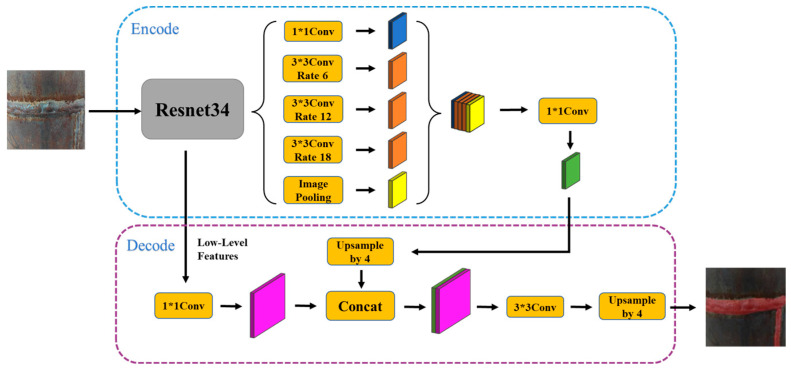
DeepLabv3+network structure.

The DeepLab semantic segmentation network based on cavity convolution was proposed to address issues, such as the sharp decline in image resolution and the loss of original information caused by continuous pooling or downsampling operations in full convolutional segmentation networks. As the latest version of the DeepLab series, the DeepLabv3+ semantic segmentation network [31] has exhibited excellent performance in multiple datasets. As shown in Figure 7, the DeepLabv3+ network comprises two main components: Encode and Decode.

The fundamental principle behind the DeepLabv3+ semantic segmentation network model is to extract and fuse image features in the Encode phase, followed by feature map analysis in the Decode phase. The DeepLabv3+ network’s performance advantage lies in the ASPP module, which combines cavity convolution with feature pyramid pooling to extract and fuse multiscale image feature information.

**Figure 8:**
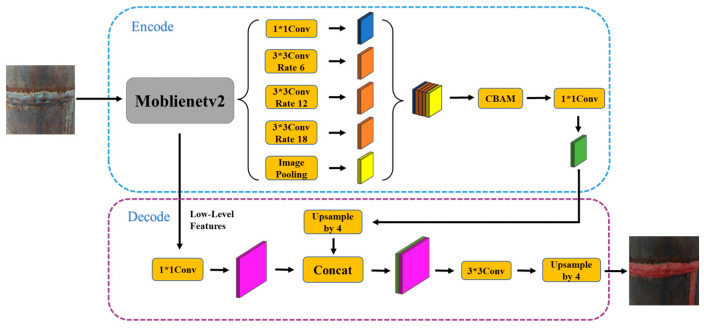
Improved DeepLabv3+ network structure.

In this study, the DeepLabv3+ model was improved from two aspects. On the one hand, an attention mechanism was introduced to improve the information extraction ability of low-resolution feature maps. On the other hand, the network model was optimized to reduce its complexity, resulting in the improved network structure shown in Figure 8.

#### 3.2.2. Introduction of Lightweight Attention Mechanism Convolutional Block Attention Module

**Figure 9:**
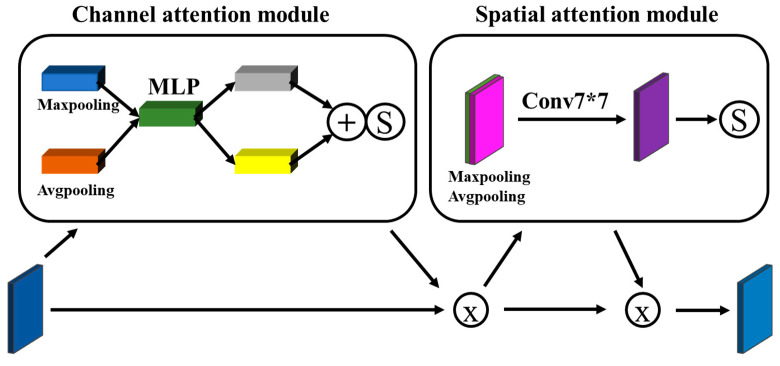
CBAM structure diagram.

The CBAM is a modular hybrid attention mechanism that was first proposed by Woo et al. [32]. It calculates attention weights from two independent dimensions, channel and space, and then applies these weights to adjust the input feature map adaptively. The structure of CBAM is shown in Figure 9.

The channel and spatial attention modules of CBAM are responsible for filtering the extracted features, ensuring the retention of important information during the encoding stage to enhance segmentation. The channel attention mechanism focuses on learning channel-specific information that greatly influences the network, and the spatial attention mechanism captures position relationship information to improve the network’s learning ability. The network can effectively learn the characteristics of weld seam images and suppress redundant information by incorporating CBAM into the encoding module of the DeepLabv3+ model, thereby facilitating the extraction of edge features of weld seams.

#### 3.2.3. Lightweight Improvement of the Model

Since semantic segmentation needs to deal with the classification of each pixel, it requires more comprehensive feature extraction and representation learning of the input image. This requires the use of more convolution operations to extract features in the image and to abstract and represent those features layer by layer in order to classify them at each pixel. In addition, semantic segmentation typically requires processing smaller input images, so the network typically requires deeper convolutional layers to extract sufficient high-level semantic features. The goal of lightweight network design is to reduce model parameters and complexity while maintaining accuracy. The DeepLabv3+ semantic segmentation network used in this study obviously improved detection accuracy, but it had a model weight of 308 M, making it unsuitable for deployment on the hardware platform of the wall-climbing robot. In this study, the backbone network and convolution module of the network model were lightweighted.

(1) Structural design of the backbone network

**Figure 10:**
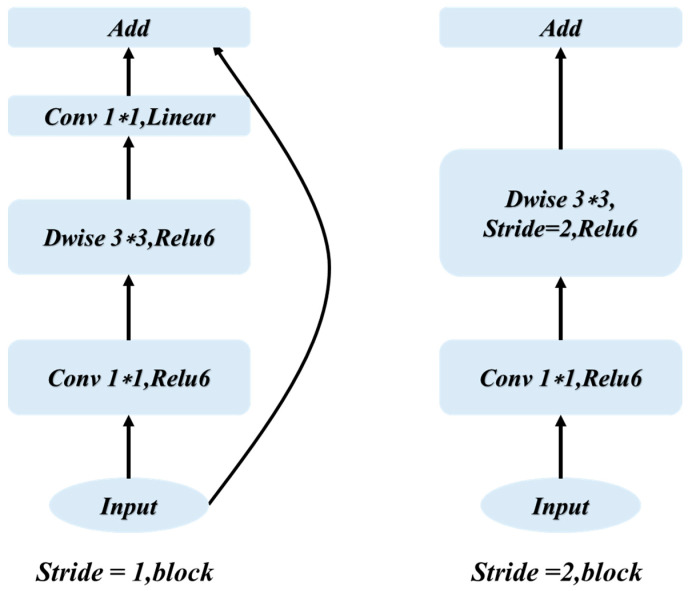
Network structure diagram of Moblienetv2.

The original DeepLabv3+ model employed Resnet [33] networks as the backbone network for feature map extraction. Although Resnet has the advantage of improving accuracy, it consists of stacked residual structures, resulting in an extremely deep network with a large number of parameters and computations. In this study, therefore, the original deep backbone network was replaced with Mobilenetv2 [34] which has a lighter network structure, as displayed in Figure 10.

(2) Design of the convolution module

After Moblienetv2 replaced the original deep backbone network, the model was successfully lightened. However, its size still did not meet the requirements of the hardware platform for embedded devices. On the basis of the improved backbone network, cavity depthwise separable convolution was introduced. All the traditional 3 × 3 convolutions were replaced with cavity depthwise separable convolutions, effectively reducing the number of parameters and computations.

The cavity depthwise separable convolution involves separating the channel and spatial dimensions in the feature map using separable convolutions. Compared to the traditional convolution kernel, hole convolution can greatly reduce the number of network parameters. This is because the hole convolution uses zero padding arranged in intervals to replace the traditional continuous convolution operation, thus avoiding a lot of parameter calculation and storage. Because the convolution operation of hole convolution can be realized via interval filling, sparse convolution can be used to speed up the training and reasoning process of the network. This can greatly improve the computing efficiency and reduce the consumption of computing resources. Depthwise separable convolution consists of two parts: depthwise convolution and pointwise convolution. The input feature map size is set to $D_{i}\times D_{i}$, the number of channels is N, the size of the standard convolution kernel is $D_{j}\times D_{j}$, and the number of output feature map channels is K. The calculation process of the standard convolution kernel, $Q_{1}$, is as follows:

(3) $$Q_{1}=D_{i}\times D_{i}\times N\times K\times D_{j}\times D_{j}$$

The calculation process of the depthwise separable convolution kernel, $Q_{2}$, is as follows:

$$Q_{2}=D_{i}\times D_{i}\times N\times D_{j}\times D_{j}+N\times K\times D_{i}\times D_{i}$$

The ratio of $Q_{2}$ to $Q_{1}$ is

(4) $$\frac{Q_{2}}{Q_{1}}=\frac{D_{i}\times D_{i}\times N\times D_{j}\times D_{j}+N\times K\times D_{i}\times D_{i}}{D_{i}\times D_{i}\times N\times K\times D_{j}\times D_{j}}=\frac{1}{K}+\frac{1}{D_{j}^{2}}$$

On the basis of the above formulas, the depthwise separable convolutions require fewer computations. The receptive field of the convolution kernel can be enlarged at the same computational cost by controlling the expansion rate, allowing for the extraction of multiscale contextual information as much as possible.

### 3.3. Welding Path Fitting

#### 3.3.1. Weld Seam Edge Detection

After the weld seam was segmented and detected by the semantic segmentation network model, the foreground segmentation image of the weld seam was obtained. However, this image alone cannot provide sufficient information for weld seam tracking and tracing by the wall-climbing robot. Hence, further processing of the welding zone image is required. The Sobel operator edge detection algorithm is relatively simple and has high detection efficiency, which is a combination of Gaussian smoothing and differential operation. The principle of the Sobel operator edge detection algorithm is to use the discrete difference operator to calculate the gray approximation of the image brightness function. Although it is more efficient than the Canny edge detection operator in practical application, its accuracy is not as good as that of Canny detection. Weld detection requires high accuracy. In this study, the edge information of the weld seam was extracted using the Canny operator. The Canny algorithm performed denoising and smoothing of the weld seam image using an appropriate Gaussian function based on rows and columns as follows,

(5) $$\begin{array}{c} G\left(x\right)=\frac{1}{\sqrt{2\pi }\sigma }e^{\frac{-x^{2}}{2\sigma^{2}}} \end{array}$$

where σ is the standard deviation of the Gaussian curve, controlling the smoothness of the result.

Next, the gradient amplitude, M(x,y), and gradient direction, H(x,y), of the image I(x,y) were calculated using the finite difference of the first-order partial derivatives of 2 × 2 adjacent areas, that is

(6) $$\begin{array}{c} M\left(x,y\right)=\sqrt{K_{x}^{2}\left(x,y\right)+K_{y}^{2}\left(x,y\right)} \end{array}$$

(7) $$\begin{array}{c} \begin{array}{c} H\left(x,y\right)=arctan\left(\frac{K_{x}\left(x,y\right)}{K_{y}\left(x,y\right)}\right) \end{array} \end{array}$$

(8) $$\begin{array}{c} f_{x}=\left[\begin{array}{cc} -0.5 & 0.5\\ -0.5 & 0.5 \end{array}\right],f_{y}=\left[\begin{array}{cc} 0.5 & 0.5\\ -0.5 & -0.5 \end{array}\right] \end{array}$$

where $K_{x}$ is the derivative value of the image pixel in the x direction. Similarly, $K_{y}$ is the derivative value of the image pixel in the y direction; $\left(x,y\right)$ is the image pixel; $f_{x}\;$ and $f_{y}$ are matrices of partial derivatives of the image in the x and y directions, respectively.

Using the above formula, the Canny edge detection operator can be used to detect the edges of the weld seam image, thereby enriching the edge detail information of the weld seam.

#### 3.3.2. Fitting of the Weld Centerline

After obtaining the weld edge information from edge detection, the least squares method is used to regress the weld edge curve and obtain the final weld center line. This centerline is then used for path planning by the wall-climbing robot.

The objective function for least square cubic polynomial fitting can be expressed as

(9) $$\begin{array}{c} A_{1}\left(x_{1},y_{1}\right),A_{2}\left(x_{2},y_{2}\right),\ldots ,A_{n}\left(x_{n},y_{n}\right) \end{array}$$

where *A* is the coefficient matrix; $x_{1},y_{1},\cdots ,x_{n}$, and $y_{n}$ represent the independent and dependent variables, respectively.

Therefore, the cubic polynomial obtained through least squares can be expressed as

(10) $$A_{1}\left(x_{1},y_{1}\right)A_{2}\left(x_{2},y_{2}\right)\cdots A_{n}\left(x_{n},y_{n}\right)=\;\begin{array}{c} \frac{A_{1}\left(x_{1},y_{1}\right)+A_{2}\left(x_{2},y_{2}\right)+\cdots +A_{n}\left(x_{n},y_{n}\right)}{n+1} \end{array}$$

where n+1 represents the number of terms in the polynomial. The optimal solution was obtained using the least squares regression approach with $A_{1}\left(x_{1},y_{1}\right)A_{2}\left(x_{2},y_{2}\right)\ldots A_{n}\left(x_{n},y_{n}\right)$. The wall-climbing robot can obtain path information for the better tracking of weld seams by fitting the centerlines of weld seams to the set of continuous data points representing straight and curved weld seams.

## 4. Experimental Results and Analysis

**Figure 11:**
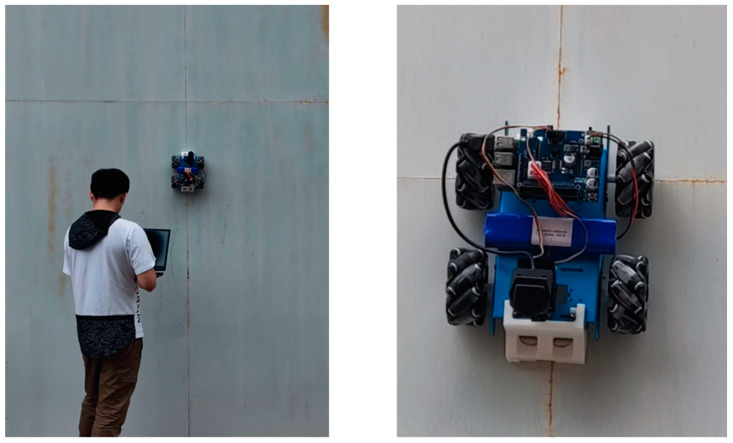
Experimental environment of the wall-climbing robot.

The experimental scene in this study involved a metal welded wall surface with a diameter of 4000 mm, a height of 2800 mm, and a thickness of 10 mm, as shown in Figure 11. Weld seams were distributed on the wall surface for the robot to identify and track. This process was remotely controlled by a researcher using a computer, who started the robot and observed its movement trajectory and weld detection status in real time.

### 4.1. Feasibility Analysis of the Wall-Climbing Robot’s Tracking and Detection

In this study, the model was optimized using the stochastic gradient descent method (signal growth deposition) as the optimizer. The dichotomic cross-entropy loss function (CE_Loss) served as the loss function for model training. The initial learning rate (*lr*) was set to 1 × 10^−2^, the weight loss ratio (weight_decay) was set to 1 × 10^−4^, the batch size (Batchsize) was set to 16, and the momentum gradient descent (momentum) was set to 0.9. In total, 500 training iterations were performed in the experiment.

**Figure 12:**
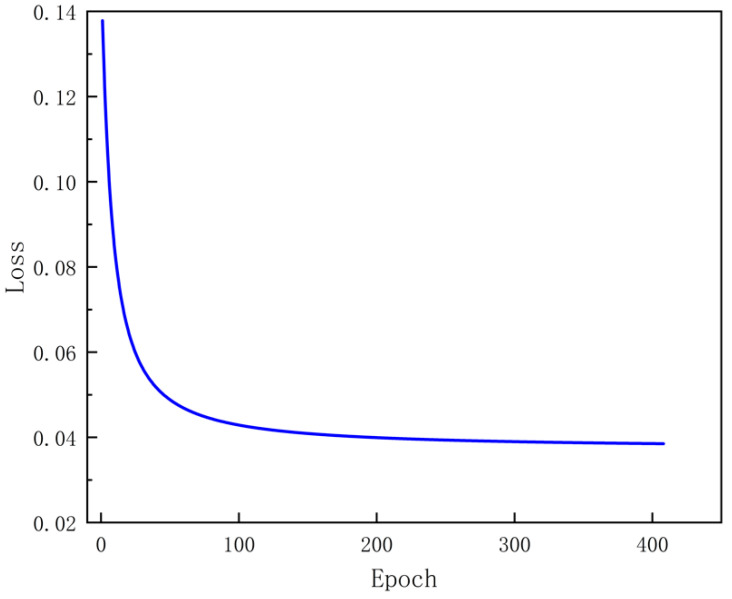
Training results.

The input image size for model training was set to 380 × 380 × 3. During training, the model weights were saved every 100 iterations, and the model was verified after each training iteration. The number of early_stop rounds was set to 100. The loss curve is displayed in Figure 12.

**Figure 13:**
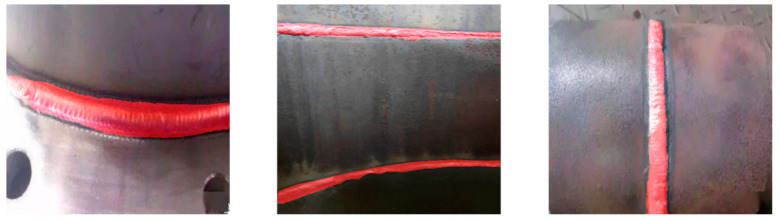
Detection results of differently shaped weld seams under different scenes using the improved algorithm.

As shown in Figure 12, the model converged quickly after 30 training iterations and then gradually descended toward the convergence edge until achieving stable convergence. This indicates that the model performed well on the validation set and gradually learned the weld seam characteristics on the training set. The accuracy of the model also improved rapidly and stabilized with training, manifesting good learning effects on the training set. The reduction in curve fluctuations during the training process suggests a reduced possibility of overfitting. In addition, the improved algorithm was used to detect weld seams of different shapes. The detection results are shown in Figure 13. The red regions in the figure represent the segmentation results obtained via the improved algorithm in this study. The detection results demonstrate the improved algorithm’s capability to achieve excellent results in various scenes, backgrounds, and types of weld seams.

### 4.2. Model Comparison

Three groups of comparative experiments were conducted to verify the performance advantages of the improved algorithm in weld seam detection. The improved DeepLabv3+ algorithm in this study was compared with the mainstream segmentation detection models a U-Net [35] and Pspnet [36] and the original Deeplabv3+ [31] model before improvement. The comparison focused on six key indexes: mIOU, pixel accuracy (PA), giga floating point operations per second (GFlops), weight file size (Weight), frames per second (FPS), and reasoning speed (Time). The results of the comparative experiments are listed in Table 3.

The two other semantic segmentation network models compared in Table 3 and the original DeepLabv3+ model all used CE_Loss as the loss function for model training, and other training parameters were kept consistent. Compared to the Pspnet network model, the improved model in this study achieved a 1.3% increase in accuracy, a 0.2% increase in mIOU, a 93.8% reduction in GFLOPs, an 87.5% reduction in model weight, a 23.5% increase in FPS, and a 20.3% reduction in single-image reasoning time. Compared to U-net semantic segmentation, the improved model demonstrated a 2.3% improvement in model accuracy, a 0.5% increase in mIOU, a 96.9% reduction in GFLOPs, a 90.5% reduction in model weight, a 50% increase in FPS, and a 32.8% reduction in single-image reasoning time. Compared to the original model DeepLabv3+, the improved model showcased a 3.2% increase in model accuracy, a 1.4% increase in mIOU, a 91.5% reduction in GFLOPs, a 92.9% reduction in model weight, a 16.7% increase in FPs, and an 11.3% reduction in single-image reasoning time.

On the basis of the above data analysis, the improved model in this study significantly reduced the computational requirements and greatly improved the calculation and reasoning speed while maintaining high accuracy. It exhibited clear advantages compared to the current mainstream semantic segmentation models in terms of weld seam detection speed.

### 4.3. Welding Path Fitting Results

**Figure 14:**
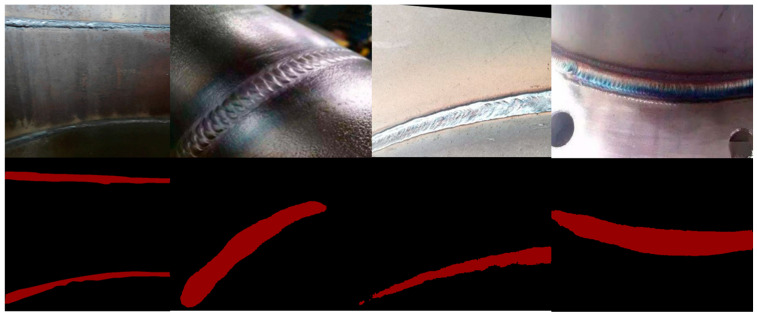
Original weld seam image and its foreground segmentation image.

Various complex scenes containing weld seams, such as inside pipelines, were selected as the experimental subjects to evaluate the performance of the welding path fitting algorithm in the weld seam tracking and detection model of this study. The original drawings of weld seams and their foreground segmentation results are displayed in Figure 14. The original images included straight and curved weld seams, and weld seams on the inner walls of pipelines. After reasoning by the improved DeepLabv3+ semantic segmentation model, the foreground segmentation image successfully separated the weld seam from the complex background, providing regional position information of the weld seam and the main part of the weld seam contour.

**Figure 15:**
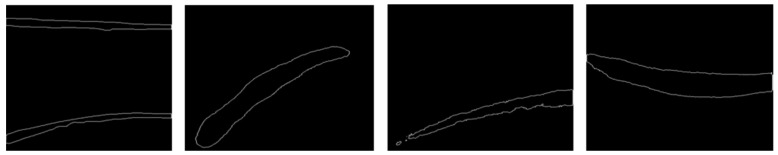
Edge detection results of weld seams.

However, the foreground segmentation image did not clearly show the edge details of the weld seam. The weld seam foreground segmentation image was subjected to edge detection using the Canny operator to obtain clearer edge details for weld seam tracking. The detection results are displayed in Figure 15.

**Figure 16:**
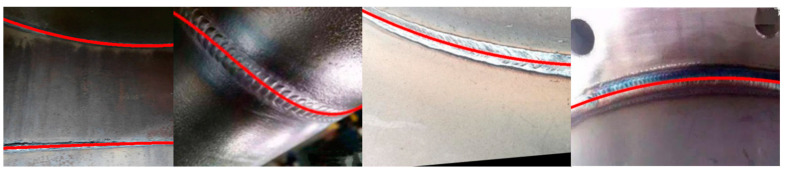
Fitting results of weld seam centerlines.

Once the contour map of the weld seam edge was obtained through edge detection, the centerlines of the weld seams were fitted to provide position information for weld seam tracking by a wall-climbing robot. In this study, the centerlines of weld seam edge contours were subjected to fitting using the least squares regression method, utilizing a quadratic polynomial for fitting. The fitting results are displayed in Figure 16.

The path fitting process shown in Figure 14, Figure 15 and Figure 16 demonstrated that the improved model in this study exhibited excellent performance from segmentation to path fitting. The single-image processing time was 71 ms, achieving a FPS of 14, meeting the real-time requirements of embedded devices. The test results in Table 4 indicate that the test dataset used to evaluate the model included 300 weld seam samples, out of which 30 had fitting errors, and 270 were fitted accurately. The fitting accuracy reached 90%. The improved model in this study demonstrated high recognition accuracy and a high reasoning speed for weld seams in complex scenes, including those with curved weld lines. The accuracy met the industrial requirements for weld seam quality inspection, and the reasoning speed satisfied the real-time performance requirements of industrial applications.

## 5. Discussion

### 5.1. Ablation Experiment

Four groups of experimental improvement schemes were designed to explore the influence of the improved model in this study on the performance of DeepLabv3+. The model training parameters for all four schemes were consistent. The influence of these schemes on model performance is shown in Table 5. Model 1 represents the original DeepLabv3+ model. Model 2 is the improved model after integrating the hybrid attention mechanism, CBAM. Model 3 replaces the feature extraction network of Model 2 with Mobilenetv2. In Model 4, all traditional 3 × 3 convolution modules are replaced by cavity depthwise separable convolution modules based on Model 3.

According to Table 5, Model 1 achieved a pixel accuracy of 97.1%, GFLOPs of 38.8, a model size of 308 Mb, a FPS of 18, and an average processing time per image of 54 ms. The original model exhibited a low calculation speed and had a model size that was unsuitable for mainstream embedded devices. The PA of Model 2 increased by 1.6% after integrating the hybrid attention mechanism, CBAM, indicating that the attention mechanism improved the model’s attention to weld seams. However, the reasoning speed decreased by 3 ms compared to that of the original model, and the FPS dropped from 18 to 17, reflecting that the introduction of the attention mechanism increased the computational load. Considering the lightweight nature of the network structure, Model 3 replaced the original Resnet34 deep network with a lighter Mobilenetv2 network as the backbone for feature extraction. The results showed that Model 3 reduced the computational complexity from 38.8 to 14.6, decreased the model size from 308 Mb to 44.6 Mb, accelerated the reasoning speed by 4 ms, and improved the FPS by 2. The pixel accuracy, PA, was maintained at 98.7% while reducing model and computational complexity. In the final model, Model 4, all traditional 3 × 3 convolution modules in the improved model were replaced by cavity depthwise separable convolution modules. The GFLOPs of the final model decreased to 3.3, the model size was 21.8 Mb, the FPS increased to 21, and the single-image reasoning time was reduced to 47 ms. This demonstrates that Model 4 achieved a balance between model accuracy and speed, resulting in optimal model performance.

The results of the ablation experiments proved that the process of the improved model in this study was reasonable. It met the requirements for seam tracking and detection while meeting the real-time requirements of embedded devices.

### 5.2. Data Augmentation Experiment

In this study, datasets were enriched using the copy and paste data augmentation strategy. This approach improved the segmentation accuracy of the model and augmented its generalization ability and robustness but also reduced the risk of overfitting during model training. A group of comparative experiments was designed to verify the effectiveness of data augmentation. Two scenarios were compared: training the model using the original image dataset without data augmentation and training the model using the augmented datasets; the results are shown in Table 6.

The improved network in this study was trained using both the data set with data augmentation and the data set without data augmentation. Table 5 shows the comparison results, considering three evaluation indexes, namely, pixel accuracy PA, average intersection ratio, mIOU, and verification loss. The augmented data set achieved a 0.4% improvement in pixel accuracy compared to that of the original data set, and the mIOU remained the same at 87.9%. The model in this study demonstrated high pixel accuracy and an average intersection ratio. Compared to the model trained without data augmentation, the model trained with data augmentation exhibited a greater decrease in verification loss, indicating better performance. This demonstrates that data augmentation aids in model convergence and effectively reduces the risk of overfitting.

## 6. Conclusions

This study focused on designing a weld seam-tracking robot that combines wall climbing capabilities with path planning using artificial intelligence technology. A welding seam-tracking detection robot was developed in this study, which consists of a permanent magnet array adsorption module, McNamun wheel and detection module. Through the uniquely designed permanent magnet adsorption module, the adsorption center of the car body can be adjusted adaptively when crossing obstacles, and the functions of climbing walls and crossing obstacles at high altitudes can be realized. This study also proposes an algorithm for seam identification and path planning based on artificial intelligence technology. Data augmentation techniques were employed to expand the dataset, enabling the trained weld segmentation model to handle a wider range of detection scenarios. The size of the Deeplabv3+ segmentation model was reduced by 92.9% after lightweight improvements, making it more suitable for embedding in hardware devices. In addition, the improved model achieved a 1.4% increase in accuracy compared to that of the original model. The application of least squares polynomials for fitting the weld segmentation results demonstrated excellent performance in accurately fitting various complex welds. The total reasoning time, from image segmentation to path fitting, was 71 ms per image, meeting the operational requirements of the weld seam tracking and detection robot. The development of this robot system has enabled intelligent detection capabilities. In future work, a broader range of weld seam types will be explored, and the application scope of the robot system will be expanded.

## Figures and Tables

**Table 1:** Performance parameters of permanent magnet.

Materials	Trademark	Remanence Induction Intensity (mT)	Shape Parameter (mm)	Coercivity (kA/m)	Innate Coercivity (kA/m)	Maximum Magnetic Energy (kJ/m^3^)
NdFeB	N52	1430–1480	$30^{2}\times \pi \times 5$	>796	>876	398–422
NdFeB	N52	1430–1480	60×40×10	>796	>876	398–422

**Table 2:** Robot performance parameters.

Symbols	Meanings	Unit	Quantitative Values
$M_{G}$	Self-weight	Kg	1.70
$M_{l}$	Maximum payload	Kg	1.96
$V_{max}$	Maximum velocity	m/s	0.2
F	Adsorption force	N	366.12
$h_{m}$	Obstacle surmounting height	Mm	11
T	Working time	Min	100

**Table 3:** Comparison of different segmentation models.

Models	mIOU/%	PA/%	GFLOPs/M	Weight/Mb	FPS	Time/ms
Pspnet	89.80	98.30	53.6	175	17	59
U-net	88.80	98	88.1	229	14	70
DeepLabv3+	87.90	97.10	38.8	308	18	53
**Ours**	**91.10**	**98.50**	**3.3**	**21.8**	**21**	**47**

**Table 4:** Test results.

Model	Data	Mistake Fitting	Current Fitting	Time	FPS
Improve model	300	30	270	71 ms	14

**Table 5:** Comparison of ablation experimental results.

Models	CBAM	Mobilenetv2	Conv	PA	GFLOPs	Weight	FPS	Time
model1				97.1%	38.8	308 Mb	18	54 ms
model2	√			98.7%	38.8	308 Mb	17	57 ms
model3	√	√		98.7%	14.6	44.6 Mb	19	53 ms
**model4**	**√**	**√**	**√**	**98.5%**	**3.3**	**21.8 Mb**	**21**	**47 ms**

**Table 6:** Comparison of data augmentation experiments.

Experiments	PA/%	mIOU/%	Loss
Model trained using image data set without data augmentation	96.7	87.9	0.096
Model trained using image data set with data augmentation	97.1	87.9	0.092

## Data Availability

All data included in this study are available upon request via contacting the corresponding author.
